# Involvement of ER stress, PI3K/AKT activation, and lung fibroblast proliferation in bleomycin-induced pulmonary fibrosis

**DOI:** 10.1038/s41598-017-14612-5

**Published:** 2017-10-27

**Authors:** Han-Shui Hsu, Chen-Chi Liu, Jiun-Han Lin, Tien-Wei Hsu, Jyuan-Wei Hsu, Kelly Su, Shih-Chieh Hung

**Affiliations:** 1 Division of Thoracic Surgery, Department of Surgery, Taipei Veterans General Hospital, Taipei, Taiwan; 2 Institute of Emergency and Critical Care Medicine, National Yang-Ming University School of Medicine, Taipei, Taiwan; 3 Division of Trauma, Emergency Department, Taipei Veterans General Hospital, National Yang-Ming University School of Medicine, Taipei, Taiwan; 4 Institute of Biomedical Sciences, Academia Sinica, Taipei, Taiwan; 5 Integrative Stem Cell Center, Department of Orthopedics, China Medical University Hospital, Taichung, 40447 Taiwan; 6 Graduate Institute of New Drug Development, Biomedical Sciences, China Medical University, Taichung, 40402 Taiwan

## Abstract

Pulmonary fibrosis is characterized by fibroblast proliferation and extracellular matrix remodelling, leading to respiratory insufficiency. The mechanisms underlying this progressive and devastating disease remain unclear. Conditions that can impair the function of the endoplasmic reticulum (ER) cause accumulation of unfolded or misfolded proteins, resulting in ER stress and activation of the unfolded protein response (UPR). ER stress has been implicated in many conditions including cancer, diabetes, obesity, and inflammation. It is also involved in lung fibrosis, through myofibroblastic differentiation of fibroblasts; however, the precise role of ER stress in lung fibrosis is unknown. The current study aimed to investigate the underlying mechanisms of ER stress inhibitors in the treatment of bleomycin-induced lung fibrosis. We demonstrated that bleomycin can activate ER stress associated proteins, including GRP78, CHOP, and ATF-4, both *in vitro* and *in vivo*. PI3K/AKT acts upstream of ER stress to affect lung fibroblast proliferation, resulting in bleomycin-induced pulmonary fibrosis. Treatment with ER stress inhibitors or a PI3K inhibitor caused a reduction in fibroblast proliferation and improved pulmonary function. The relationship between PI3K/AKT/mTOR and ER stress in pulmonary fibrosis, and the application of PI3K inhibitors and ER stress inhibitors in the treatment of pulmonary fibrosis require further investigation.

## Introduction

Pulmonary fibrosis is characterized by fibroblast proliferation and extracellular matrix remodelling, leading to respiratory insufficiency. The most common form of pulmonary fibrosis is idiopathic pulmonary fibrosis (IPF), a chronic pulmonary disease of unknown origin with poor prognosis due to ineffective treatments^1,2^. Many mechanisms are involved in the pathogenesis of IPF, such as epithelial cell injury with activation of interstitial inflammation, and fibroblast proliferation with extracellular matrix collagen deposition^3^. However, the mechanisms that underlie this progressive and devastating disease are still not clear.

Bleomycin was once used as an antineoplastic agent, but is now thought to cause dose-dependent interstitial pulmonary fibrosis^4^. Intratracheal administration of bleomycin to the lungs of rodents has been shown to cause alveolar cell damage, an inflammatory response, epithelial-mesenchymal transition (EMT), fibroblast proliferation and subsequent extracellular matrix deposition, all of which resemble human fibrotic lung disease^5^. Bleomycin-induced pulmonary fibrosis is the most commonly used animal model of idiopathic pulmonary fibrosis for studying disease pathogenesis and testing novel pharmaceutical compounds^6^.

The endoplasmic reticulum (ER) is an intracellular organelle responsible for the folding and sorting of proteins^7,8^. Various conditions that impair ER function can cause accumulation of unfolded or misfolded proteins, resulting in ER stress, which leads to activation of a signal response termed the unfolded protein response (UPR)^9^. ER stress can be reduced by UPR signalling mediated by three transmembrane ER proteins: inositol requiring ER-to-nucleus signal kinase (IRE)1, activating transcription factor (ATF)6 and double-stranded RNA-activated kinase (PKR)-like ER kinase (PERK)^10,11^. Active IRE1 can cleave x-box binding protein-1 (XBP-1) mRNA in a site-specific manner to promote unconventional splicing, generating an active transcription factor^12^. PERK is an ER stress-sensitive eukaryotic initiation factor (eIF) 2α kinase. Following ER stress, PERK phosphorylates and activates eIF2α, which attenuates cap-dependent translation^13^.

ER stress has been implicated in various diseases, including cancer, diabetes, obesity and inflammation^14,15^. ER stress is also involved in lung fibrosis through the control of fibroblast proliferation and myofibroblastic differentiation^16^. Zhao *et al*. reported that melatonin alleviates ER stress and ER stress-mediated epithelial-mesenchymal transition (EMT) in lung fibrosis^17^. The precise mechanism by which ER stress is involved in lung fibrosis remains poorly understood.

The aim of current study was to investigate the underlying mechanisms of ER stress inhibitors in the treatment of bleomycin-induced lung fibrosis. We found that bleomycin activated ER stress associated proteins, including GRP78, CHOP, and ATF-4, both *in vitro* and *in vivo*. When ER stress inhibitors or pAKT inhibitors were administered, the extent of pulmonary fibrosis reduced and pulmonary function improved.

## Results

### Activation of ER stress associated proteins in bleomycin-induced pulmonary fibrosis

**Figure 1:**
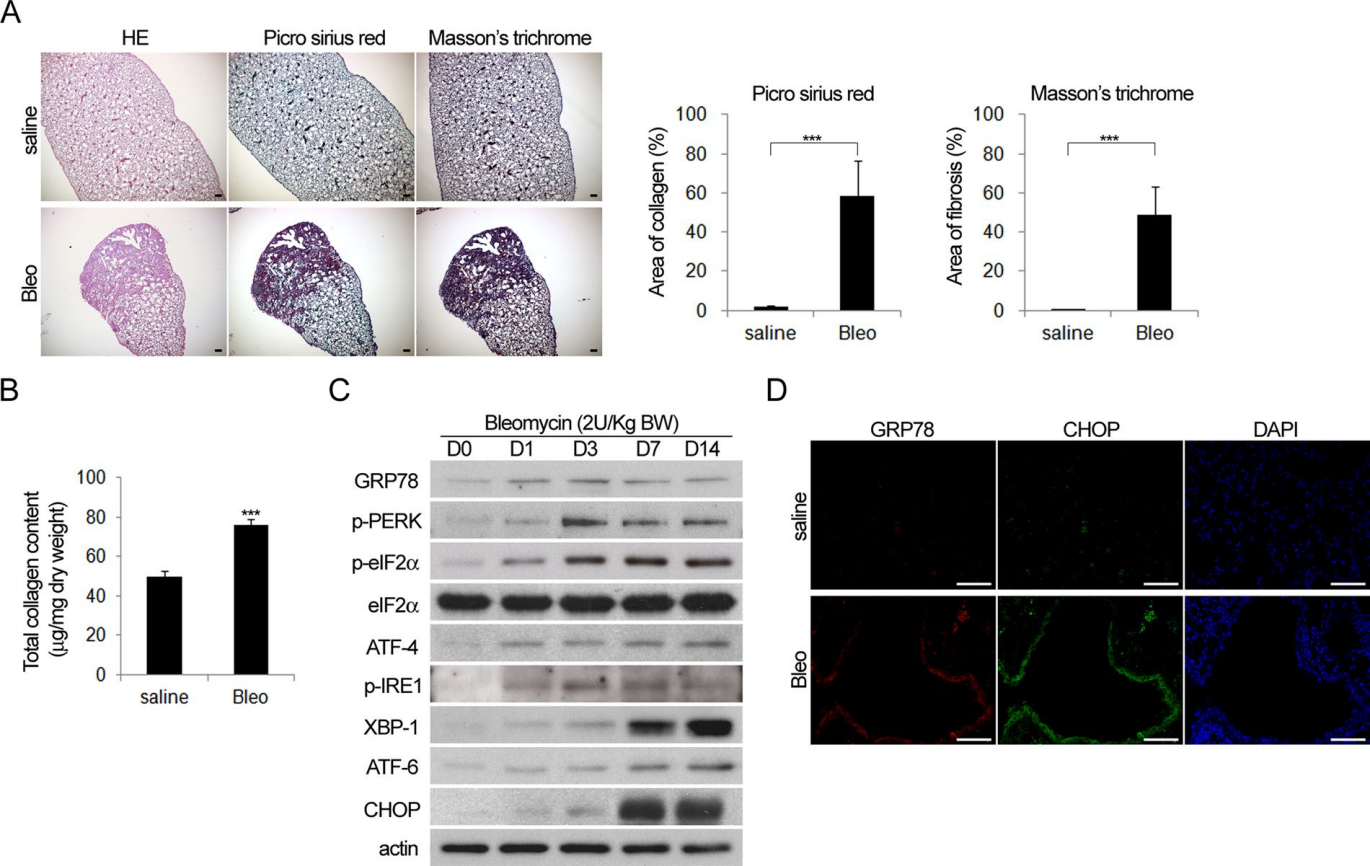
Bleomycin-induced pulmonary fibrosis was associated with ER stress activation. Mice with intratracheal administration of bleomycin (2 U/kg) or saline (vehicle) were sacrificed 14 days later and the lung specimens were harvested for (**A** Left) histological analysis with HE, Picro Sirius red and Masson’s trichrome staining followed by (**A** Right) quantification, (**B**) The results of total collagen assay, (**C**) Western blot analysis (Cropped blots are displayed; Full-length blots are presented in Supplementary Figure, labeled Figure S1), and (**D**) immunofluorescence for the expression of proteins associated with ER stress activation.

Pulmonary fibrosis was induced in mice by intratracheal administration of bleomycin. Picro sirius red and Masson’s trichrome staining confirmed increased fibrosis of lung tissue in mice following bleomycin treatment (Fig. 1A and B). Western blot analysis and immunofluorescence showed that proteins associated with ER stress, including GRP78, CHOP, ATF-4, and XBP-1, were activated in the alveolar surface of fibrotic lung tissues induced by bleomycin administration (Fig. 1C and D). These data suggest that bleomycin-induced pulmonary fibrosis increases ER stress activation.

### Treatment with ER stress inhibitors reduces the extent of pulmonary fibrosis

**Figure 2:**
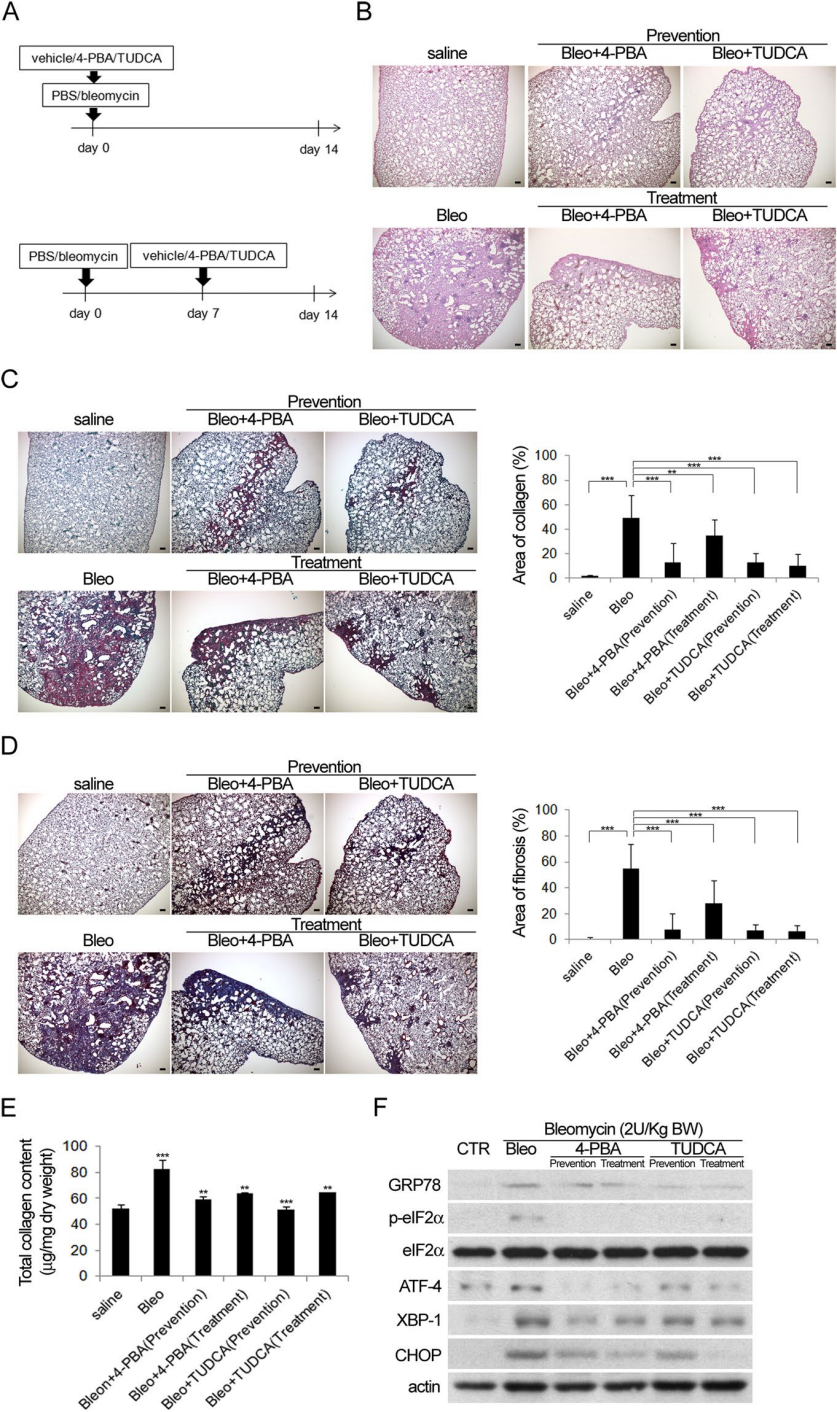
Bleomycin-induced pulmonary fibrosis was attenuated by treatment with ER stress inhibitors. (**A**) Flow chart of the experimental procedure. Mice with intratracheal administration of bleomycin (2 U/kg) or saline (vehicle) were treated with or without 4-PBA or TUDCA before (Upper: prevention) or 7 days (Lower: treatment) after bleomycin intratracheal instillation. The mice were sacrificed 14 days later and the lung specimens were harvested for histological analysis with (**B**) HE, (**C**) picro pirius red and (**D**) Masson’s trichrome staining followed by (**C** and **D** Right) quantification, (**E**) The results of total collagen assay and (**F**) Western blot analysis (Cropped blots are displayed; Full-length blots are presented in Supplementary Figure, labeled Figure S2) for the expression of proteins associated with ER stress activation.

To determine whether ER stress activation is involved in the induction of pulmonary fibrosis by bleomycin, an ER stress inhibitor, 4-PBA or TUDCA, or vehicle only was administrated on day 0 (prevention group) or day 7 (treatment group) after bleomycin treatment. Animals were sacrificed on day 14 (Fig. 2A). Lung sections from animals that received 4-PBA or TUDCA before or 7 days after bleomycin treatment showed markedly reduced pulmonary fibrosis compared to vehicle-only controls, as shown by HE (Fig. 2B), picro sirius red (Fig. 2C) and Masson’s trichrome staining (Fig. 2D). Quantitation of the areas containing collagen deposition revealed that ER stress inhibitors reduced bleomycin-induced pulmonary fibrosis, especially when administered before bleomycin treatment (Fig. 2C,D and E). Western blot analysis also showed that ER stress inhibitors blocked the ER stress activation induced by bleomycin (Fig. 2F). These data suggest that ER stress activation is involved in pulmonary fibrosis induced by bleomycin, and that treatment with ER stress inhibitors can both prevent and treat pulmonary fibrosis.

### Bleomycin activates ER stress and AKT in primary lung fibroblast cultures

**Figure 3:**
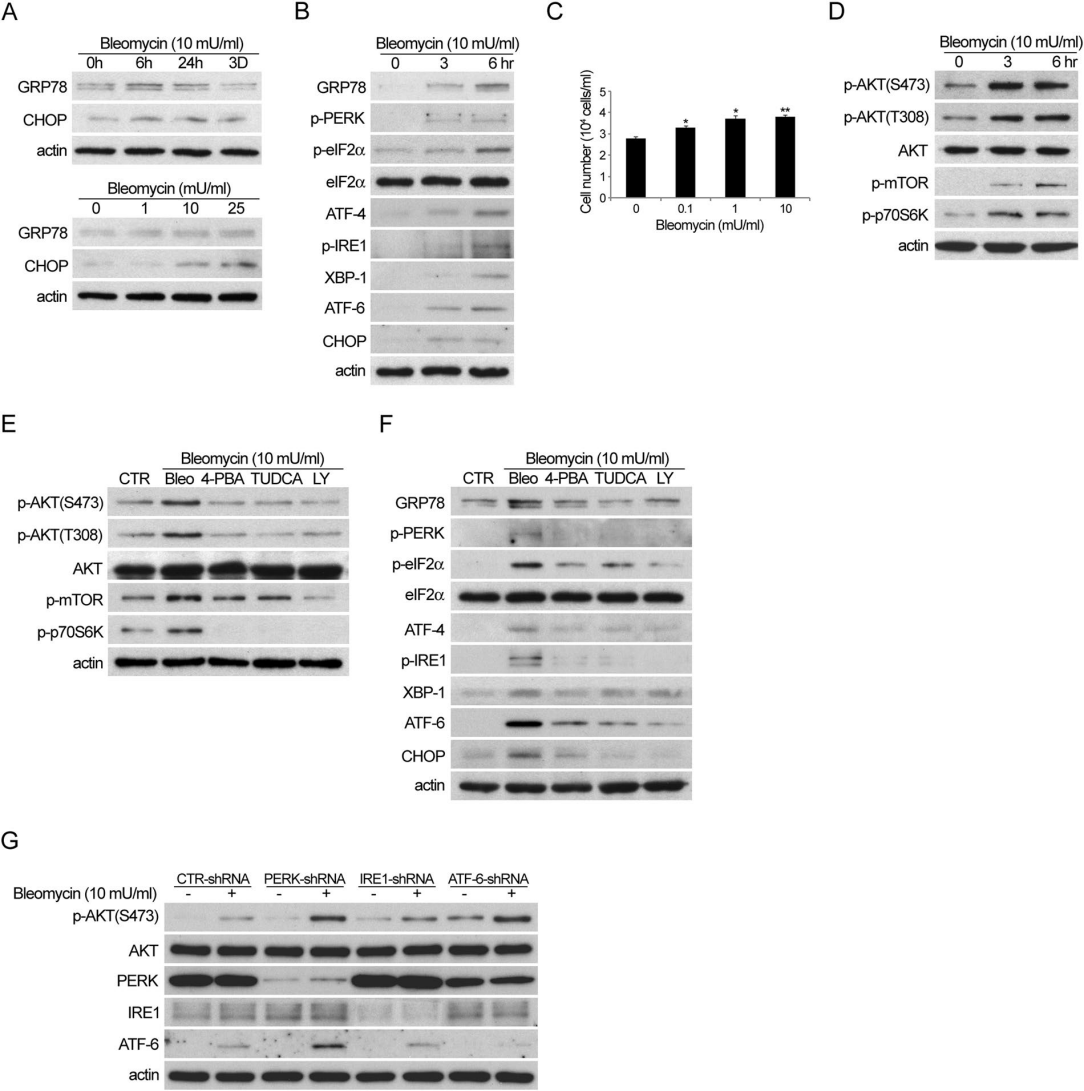
Bleomycin induced ER stress and AKT activation in murine lung fibroblast culture. (**A**–**D**) Cells before or indicated time period after treatment with indicated concentration of bleomycin were subjected to western blot analysis for the expression of proteins associated with (**A**,**B**) ER stress or (**D**) AKT activation, and (**C**) cell number counting (24 hours). (**E**–**G**) Cells were treated without (CTR) or with bleomycin in the absence or presence of ER stress or PI3K inhibitor for 6 hours, followed by western blot analysis for the expression of proteins associated with (**E**) ER stress or (**F**) AKT activation. (**G**) Cells transfected with control, PERK, ATF6 and IRE1 shRNA were treated without or with bleomycin for 6 hours, followed by western blot analysis. (Cropped blots are displayed; Full-length blots are presented in Supplementary Figure, labeled Figure S3B and C).

To further investigate the mechanisms and signalling pathways involved in bleomycin-induced pulmonary fibrosis, we used primary lung fibroblast cultures. Western blot analysis revealed that bleomycin induced the expression of GRP78 and CHOP in a time- and dose- dependent manner (Fig. 3A). Proteins associated with ER stress were activated up to 6 hours after bleomycin treatment (Fig. 3B). An increase in fibroblast proliferation was also observed when cells were treated with bleomycin (Fig. 3C). In addition, bleomycin activated PI3K/AKT and its downstream molecule, mammalian target of rapamycin (mTOR), which is important for fibroblast proliferation (Fig. 3D)^18^. When ER stress inhibitors or a PI3K inhibitor (LY294002) was added to lung fibroblasts treated with bleomycin, levels of p-AKT, p-mTOR and p-p70S6K were reduced (Fig. 3E). Furthermore, the expression of proteins associated with ER stress was also reduced when ER stress inhibitors or a PI3K inhibitor were added to lung fibroblasts treated with bleomycin (Fig. 3F). To further clarify the relationship between the PI3K/AKT/mTOR pathway and ER stress in lung fibrosis, the protein level of each molecule at some earlier time point were checked and the results showed that the expression of phosphorylated AKT was increased at 30 minutes after bleomycin was added, followed by increased expression of ER stress associated proteins including PERK, ATF6 and IRE1 at 90 minutes after bleomycin treatment (Supplementary Figure S3A). We also have used the PERK, ATF6 and IRE1 shRNA to knockdown PERK, ATF6 and IRE1 expression in the experiment and found that activation of PI3K/AKT pathway is not affected by knockdown of PERK, ATF6 and IRE1 expression (Fig. 3G). These data suggest that PI3K-mTOR pathway activates ER stress pathway in bleomycin-induced pulmonary fibrosis.

### Treatment with ER stress inhibitors reduces pulmonary fibrosis and levels of p-AKT and p-mTOR *in vivo*

**Figure 4:**
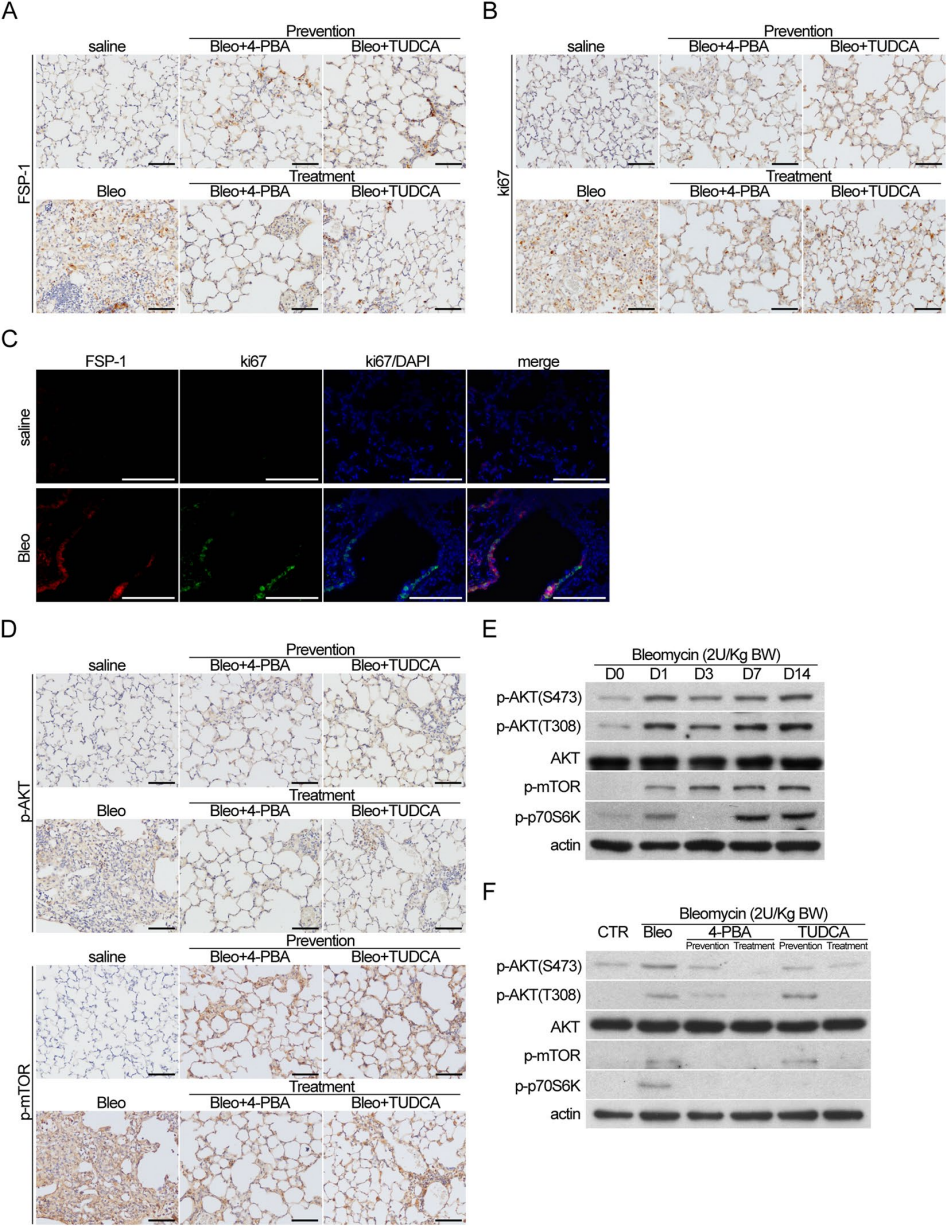
Treatment of ER stress inhibitors reduced pulmonary fibrosis and expression of p-AKT and p-mTOR *in vivo*. Mice with intratracheal administration of bleomycin (2 U/kg) or saline (vehicle) were treated with or without 4-PBA (500 mg/kg, i.p.) or TUDCA (500 mg/kg, i.p.) before (prevention) or 7 days (treatment) after bleomycin intratracheal instillation. The mice were sacrificed 14 days later and the lung specimens were harvested for (**A**,**B**,**D**) immunohistochemical analysis, (**C**) immunofluorescence, (**E**) Western blot analysis of lung harvested from Mice before or indicated time periods after intratracheal administration of bleomycin (2 U/kg) and (**F**) western blot analysis with or without treatment of 4-PBA and TUDC, (Cropped blots are displayed; Full-length blots are presented in Supplementary Figure, labeled Figure S4).

To determine whether bleomycin-induced pulmonary fibrosis is due to lung fibroblast proliferation, immunohistochemistry for FSP-1, a lung fibroblast marker, and Ki67, a cell proliferation maker, was performed on lung sections. Results showed that expression of FSP-1 and Ki67 was increased following bleomycin treatment (Fig. 4A). Furthermore, when ER stress inhibitors were added, FSP-1 and Ki67 expression was decreased (Fig. 4B). These data indicate that ER stress inhibitors can reduce cell proliferation in lung fibrosis induced by bleomycin treatment. Immunofluorescence for FSP- and Ki67 in lung sections of control and bleomycin treatment groups also showed increased Ki67 expression in FSP-1+ cells in bleomycin-induced pulmonary fibrosis (Fig. 4C), suggesting that lung fibroblast proliferation is increased in bleomycin-induced pulmonary fibrosis. Levels of p-AKT and p-mTOR were also decreased in bleomycin-induced pulmonary fibrosis following treatment with ER stress inhibitors (Fig. 4D). Western blot analysis of lung tissues showed that levels of p-AKT and p-mTOR were increased in bleomycin-induced pulmonary fibrosis from day 0 through day 14 (Fig. 4E). When treated with ER stress inhibitors, p-AKT and p-mTOR levels were decreased in both treatment and prevention groups (Fig. 4F). These data suggest that the PI3K/AKT pathway is associated with ER stress signalling and has an important role in inducing lung fibroblast proliferation in bleomycin-induced pulmonary fibrosis.

### PI3K inhibition reduces pulmonary fibrosis through inhibition of AKT and mTOR phosphorylation

**Figure 5:**
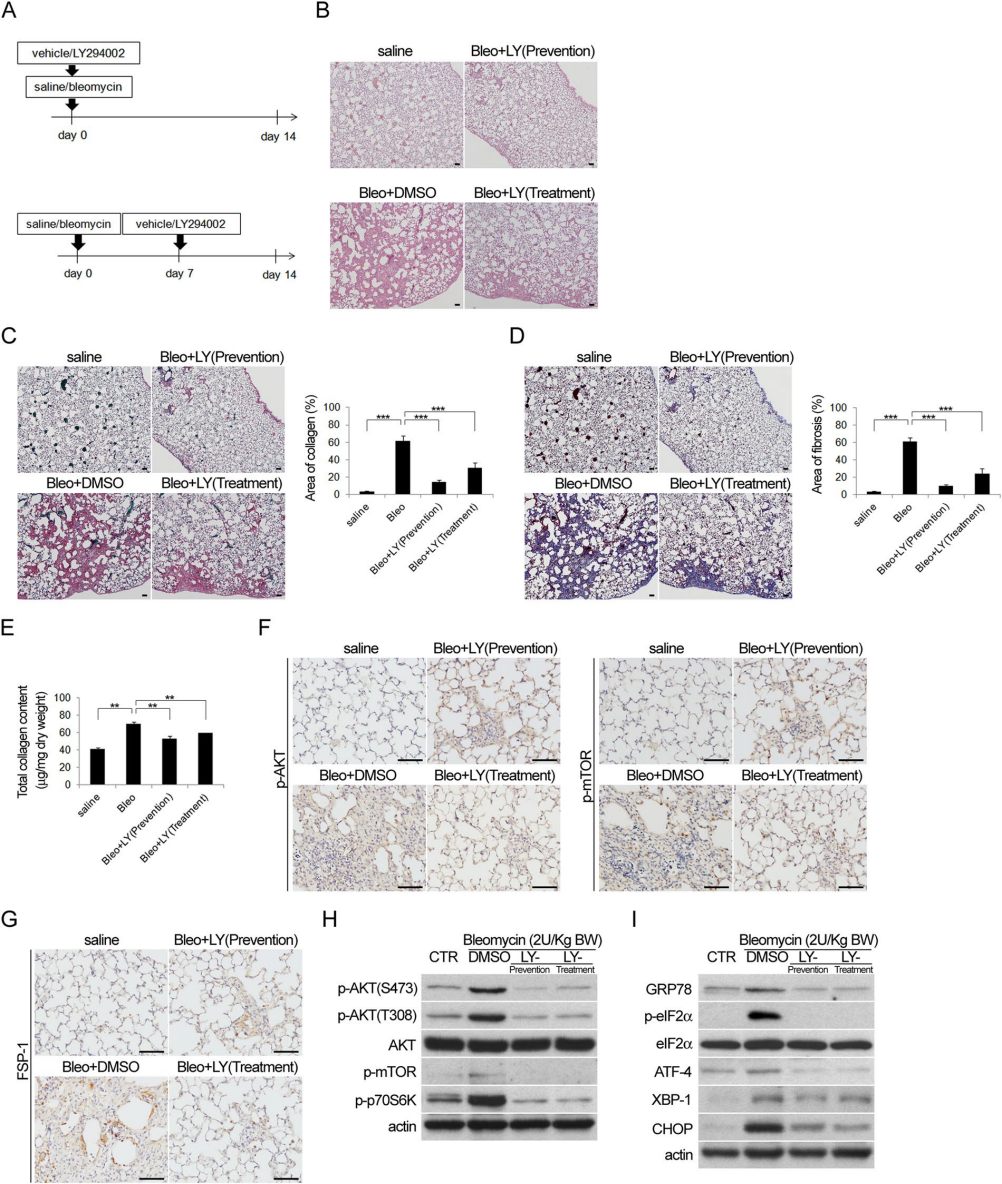
Bleomycin-induced pulmonary fibrosis was attenuated by treatment with PI3K inhibitor. (**A**) Flow chart of the experimental procedure. Mice with intratracheal administration of bleomycin (2 U/kg) or saline (vehicle) were treated with or without PI3K inhibitor, LY294002 (LY, 50 mg/kg, i.p.) before (Upper: prevention) or 7 days (Lower: treatment) after bleomycin intratracheal instillation. The mice were sacrificed 14 days later and the lung specimens were harvested for histological analysis with (**B**) HE, (**C**) picro pirius red and (**D**) Masson’s trichrome staining followed by (**C** and **D** Right) quantification, (**E**) the results of total collagen assay, (**F** and **G**) immunohistochemical analysis, and (**H**,**I**) Western blot analysis for the expression of proteins associated with (**H**) AKT and (**I**) ER stress after treatment of PI3K inhibitor, LY294002, in control, prevention and treatment groups. (Cropped blots are displayed; Full-length blots are presented in Supplementary Figure, labeled Figure S5).

To determine whether the PI3K/AKT pathway is associated with pulmonary fibrosis *in vivo*, mice with bleomycin-induced pulmonary fibrosis were treated with a PI3K inhibitor prior to bleomycin administration (prevention group), or 7 days after bleomycin was given (treatment group) (Fig. 5A). HE (Fig. 5B), picro sirius red (Fig. 5C), Masson’s trichrome staining (Fig. 5D) and total collagen count (Fig. 5E) of lung tissue sections showed that pulmonary fibrosis was reduced following treatment with the PI3K inhibitor, no matter when it was given (before or after bleomycin treatment). Immunohistochemistry also demonstrated that p-AKT and p-mTOR expression was decreased in the lungs of mice with bleomycin-induced pulmonary fibrosis following PI3K inhibitor treatment (Fig. 5F). FSP-1 expression was reduced in bleomycin-induced pulmonary fibrosis treated with the PI3K inhibitor, indicating that the inhibitor reduced pulmonary fibrosis through inhibition of fibroblast proliferation (Fig. 5G). Western blot analysis of lung tissues from mice with bleomycin-induced pulmonary fibrosis with or without PI3K inhibitor treatment showed that p-AKT and p-mTOR levels were decreased after treatment with the inhibitor (in prevent and treatment groups, Fig. 5H). Levels of ER stress-associated proteins were also decreased following treatment with the PI3K inhibitor (in prevention and treatment groups, Fig. 5I), again indicating that the PI3K/AKT pathway acts upstream of ER stress in bleomycin-induced pulmonary fibrosis.

### PTEN inhibition activates PI3K/AKT signalling and ER stress both *in vitro* and *in vivo* and results in pulmonary fibrosis

**Figure 6:**
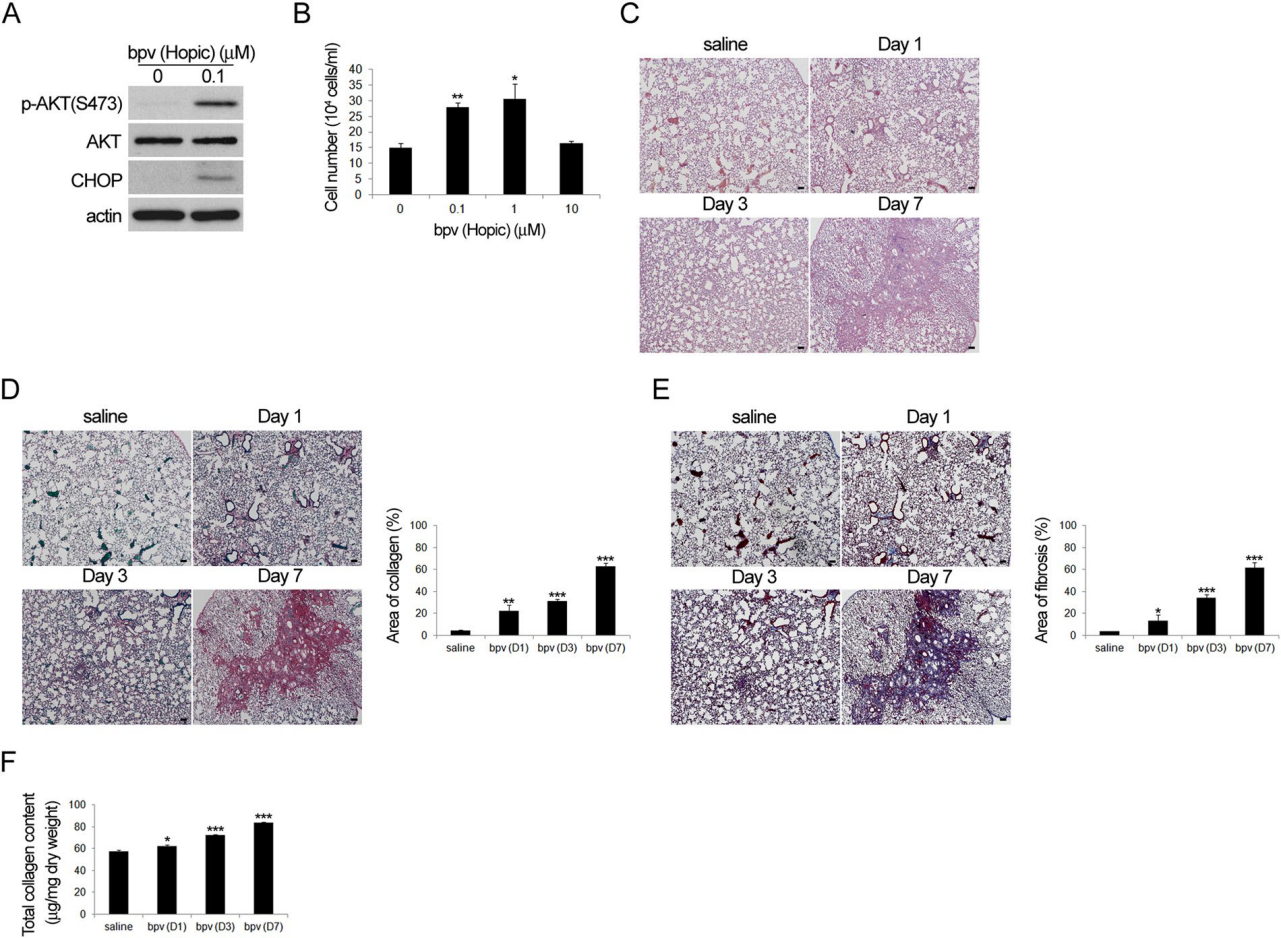
PTEN inhibitor activated the PI3K/AKT pathway in murine lung fibroblast culture and also induced pulmonary fibrosis *in vivo*. (**A**,**B**) Cells treated with or without PTEN inhibitor bpV (pic) for 24 hours were subjected to (**A**) western blot analysis (Cropped blots are displayed; Full-length blots are presented in Supplementary Figure, labeled Figure S6), and (**B**) cell number counting (24 hours). (**C**–**E**) Mice with intratracheal administration of bpV (2.5 mm) were sacrificed 1, 3 or 7 days later and the lung specimens were harvested for histological analysis with (**C**) HE, (**D**) picro pirius red and (**E**) Masson’s trichrome staining followed by (**D** and **E** Right) quantification, (**F**) The results of total collagen assay.

Having shown that the PI3K/AKT pathway is associated with bleomycin induced-pulmonary fibrosis, we further tested whether a PTEN inhibitor (bpV) could induce pulmonary fibrosis. The PTEN inhibitor was added to primary lung fibroblasts to increase p-AKT and CHOP levels (Fig. 6A). Dose-dependent enhancement of lung fibroblast proliferation was observed when up to 1 μM of bpV was added to the cells (Fig. 6B). In an animal model, pulmonary fibrosis was also induced in mice treated with a PENT inhibitor, as revealed by HE (Fig. 6C), picro sirius red (Fig. 6D), Masson’s Trichrome staining (Fig. 6E) and total collagen count (Fig. 6F). These data suggest that the PI3K/AKT pathway is essential for bleomycin-induced pulmonary fibrosis.

### ER stress or PI3K inhibitor treatment improves pulmonary function in mice with bleomycin-induced pulmonary fibrosis

**Figure 7:**
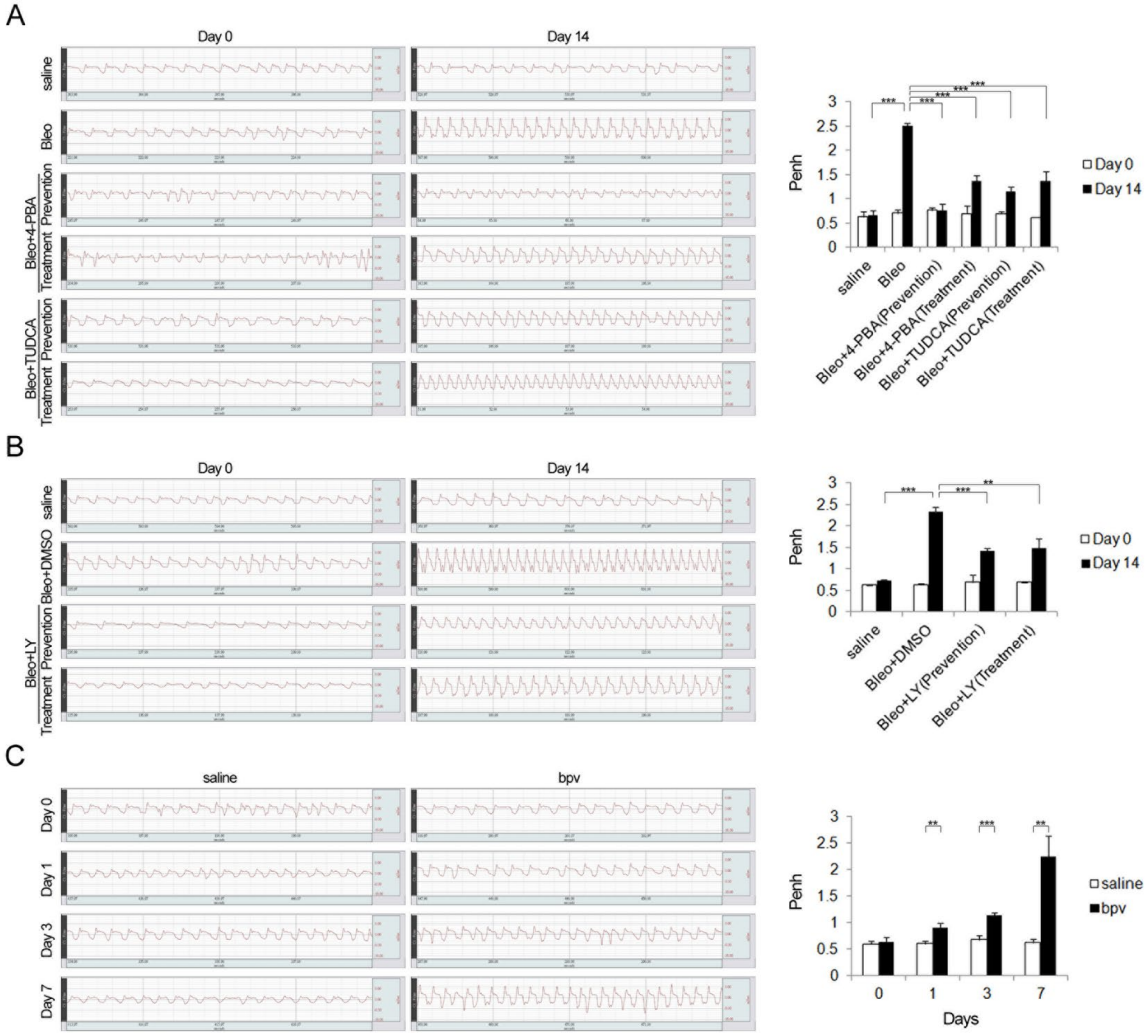
Pulmonary function in bleomycin-induced pulmonary fibrosis was improved by treatment with ER stress and PI3K inhibitors. (**A**,**B**) Barometric plethysmography was conducted in mice before or 14 days after intratracheal administration of bleomycin (2 U/kg) or saline (vehicle), treated with or without (**A**) 4-PBA or TUDCA (500 mg/kg, i.p.), or (**B**) PI3K inhibitor, LY294002 (LY, 50 mg/kg, i.p.) before (prevention) or 7 days (treatment) after bleomycin intratracheal instillation. (**C**) Barometric plethysmography was conducted in mice before or indicated time periods after intratracheal administration of bpV (2.5 mm).

Pulmonary function was analyzed in mice with bleomycin-induced pulmonary fibrosis using barometric plethysmography. The results showed improved pulmonary function in mice treated with ER stress or a PI3K inhibitor, in both prevention and treatment groups compared to vehicle alone (Fig. 7A and B). In contrast, mice treated with a PTEN inhibitor had impaired pulmonary function compared to those treated with vehicle alone (Fig. 7C). These data together suggest that PI3K and ER stress inhibitors are effective in the treatment of bleomycin-induced pulmonary fibrosis.

## Discussion

Pulmonary fibrosis is the final stage of several diffuse parenchymal lung diseases, characterized by excessive matrix deposition and lung architecture destruction, ultimately leading to respiratory insufficiency^1,2^. The pathophysiological basis of idiopathic pulmonary fibrosis has been the subject of much debate over the last few decades. Emerging new findings associated with pulmonary fibrosis, including ER stress, have also been reported^16,17^. ER stress is caused by conditions that disturb the processing and folding of proteins, which results in accumulation of unfolded protein response^9^. Baek *et al*. reported that ER stress is involved in the regulation of myofibroblastic differentiation in pulmonary fibrosis^16^. Lu *et al*. suggested that bleomycin can induce a direct fibrogenic effect on lung fibroblasts by upregulating collagen expression and cell proliferation through the PI3K/AKT pathway^19^. In the present study, we sought to evaluate the role of ER stress in bleomycin-induced pulmonary fibrosis. Using an animal model, we found that ATF-4 and eIF2α were activated immediately following bleomycin treatment, while levels of GRP78 and CHOP expression increased from day 7 through day 14. To further investigate whether an ER stress inhibitor could be used in the treatment of pulmonary fibrosis, 4-PBA and TUDCA were given to mice either before (prevention group) or 7 days after (treatment group) bleomycin treatment. Histology and immunostaining of lung sections from mice that received 4-PBA or TUDCA treatment either before or 7 days after intratracheal administration of bleomycin showed markedly reduced pulmonary fibrosis compared to that in the animals that received vehicle only. These results indicate that ER stress inhibitors could be effecting in the treatment of pulmonary fibrosis.

Tanaka *et al*. suggested that CHOP plays an important role in bleomycin-induced pulmonary fibrosis. However, the relationship between ER stress and PI3K signalling in bleomycin-induced pulmonary fibrosis was not addressed in the study^20^. To investigate the underlying mechanisms and signalling pathways involved in bleomycin-induced pulmonary fibrosis, we further used primary lung fibroblast cultures from C57BL/6 mice. The data showed that both ER stress and the PI3K/AKT/mTOR pathway are activated within 6 hours after bleomycin treatment. To elucidate the relationship between ER stress and PI3K/AKT/mTOR signalling in bleomycin-induced pulmonary fibrosis, a PI3K inhibitor, LY294002, was used to determine whether AKT/mTOR inhibition would ameliorate ER stress. We found that the PI3K inhibitor did indeed inhibit ER stress activation. These data demonstrate that PI3K/AKT acts upstream of ER stress to affect lung fibroblast proliferation, resulting in bleomycin-induced pulmonary fibrosis.

AKT is involved in the regulation of several target proteins that control cell proliferation apoptosis. mTOR, an AKT target protein, plays a critical role in cell cycle progression from G1 to S phase. The AKT/mTOR pathway is frequently abnormal in a variety of cancers, making it an attractive target for anti-cancer therapies^18^. Immunohistochemical analysis of lung biopsy specimens from IPF patients revealed that fibroblasts within fibrotic foci expressed low levels of PTEN and upregulated AKT^21^. Noh *et al*. also reported that PTEN suppression together with AKT/mTOR activation desensitizes IPF fibroblasts from collagen matrix-induced cell death^22^. The relationship between ER stress and PI3K/AKT pathway in pulmonary fibrosis has not been elucidated. Qin *et al*. reported that ER stress-induced autophagy is partly attributed to the downregulation of AKT/mTOR pathway^23^. However, Thon *et al*. demonstrated that Leptin induced GRP78 expression through the PI3K-mTOR pathway in neuronal cells^24^. In the current study, we demonstrate for the first time that the PI3K inhibitor did indeed inhibit ER stress activation. These data demonstrate that PI3K/AKT acts upstream of ER stress to affect lung fibroblast proliferation, resulting in bleomycin-induced pulmonary fibrosis.

## Conclusions

We demonstrated, in the present study, that bleomycin can activate ER stress associated proteins, including GRP78, CHOP, and ATF-4, both *in vitro* and *in vivo*. PI3K/AKT acts upstream of ER stress to affect lung fibroblast proliferation, resulting in bleomycin-induced pulmonary fibrosis. Treatment with ER stress inhibitors or a PI3K inhibitor caused a reduction in fibroblast proliferation and improved pulmonary function. The relationship between PI3K/AKT/mTOR and ER stress in pulmonary fibrosis, and the application of PI3K inhibitors and ER stress inhibitors in the treatment of pulmonary fibrosis require further investigation.

## Methods

### Animal model

Eight-week-old male C57BL/6 mice were purchased from BioLASCO Taiwan Co., Ltd. (Taipei, Taiwan). Pulmonary fibrosis was induced by intratracheal administration of 2 U/kg body weight of bleomycin (Sigma-Aldrich, St. Louis. MO) in 50 μl of sterile phosphate-buffered saline (PBS). The control group received the same volume of sterile PBS. Animals were sacrificed 14 days after bleomycin treatment, and lungs were removed for analysis. For administration of ER stress inhibitors and PI3K inhibitor, 4-PBA (200 mg/kg body weight), TUDCA (200 mg/kg body weight) or PI3K inhibitor were dissolved in PBS and administered intraperitoneally from day 0 (prevention group) or day 7 (treatment group) to each animal treated with bleomycin. Animals were sacrificed at 14 days for further studies. All animal experiments were conducted in accordance with the committee guidelines of Taipei Veterans General Hospital and approved by the IACUC (Institutional Animal Care and Use Committee of Taipei Veterans General Hospital, No. IACUC 2014-099).

### Primary cell culture

A C57BL/6 mouse (Four-week-old) lung fibroblast cell line was used for *in vitro* studies. Cells were cultured in DMEM medium supplemented with 10% foetal bovine serum (FBS) containing 100 U/ml penicillin G and 100 μg/ml streptomycin. Cells were passaged by trypsin treatment and were incubated under an atmosphere of 95% air and 5% CO_2_ at 37 °C. Cell viability was more than 95% as measured by trypan blue dye exclusion.

### HE, Masson’s trichrome, and picro sirius red staining

Lungs were fixed overnight with 4% paraformaldehyde at a constant pressure then embedded in paraffin. Sections were cut on a microtome, mounted onto slides, and stained with hematoxylin-eosin (HE), Masson’s trichrome (Sigma-Aldrich, St. Louis, MO) and picro sirius red (Sigma-Aldrich, St. Louis, MO). The area of trichrome or picro sirius red staining in a section was outlined and quantified using a light microscope attached to an image-analysis system (Image-Pro Plus; Media Cybernetics, Silver Spring, MD).

### Immunohistochemistry and immunofluorescence

Paraffin-embedded lung tissue sections were deparaffinized and rehydrated. After antigen retrieval, tissues were fixed with 2% paraformaldehyde (Sigma-Aldrich, St. Louis, MO) in PBS, and permeabilized with 0.1% Triton X-100 (Sigma-Aldrich, St. Louis, MO) in PBS. After quenching with 3% peroxide for 20 minutes, sections were blocked with PBS/0.5% BSA (blocking solution) for 20 minutes and incubated overnight with primary antibodies against Ki67 (Abcam, Cambridge, MA), FSP-1 (Abcam, Cambridge, MA), p-AKT, Ser473 (Cell Signaling Technology, Danvers, MA), p-mTOR, Ser2448 (Cell Signaling Technology, Danvers, MA) or blocking solution at 4 °C. Sections were washed extensively in PBS and incubated with biotinylated goat-anti-rabbit IgG (1:200 in blocking solution) or goat-anti-mouse IgG2b secondary antibodies in blocking buffer for one hour at room temperature. Sections were washed and further incubated with streptavidin-horseradish peroxidase (1:4000) in PBS. Antigen-antibody complexes were detected using a diaminobenzidine substrate detection kit (DAB, Vector Laboratories, Burlingame, CA). Images were obtained using an Olympus Provis AX70 microscope equipped with a digital camera and processed using Adobe Photoshop. For immunofluorescence, sections were blocked with 2% bovine serum albumin (BSA; Sigma-Aldrich, St. Louis, MO) in PBS for 1 hour, followed by incubation with primary antibodies overnight at 4 °C. Slides were then stained with Alexa Fluor 488 (green) or Alexa Fluor 546 (red) conjugated secondary antibodies (Invitrogen, Carlsbad, CA). For identification of nuclei, DAPI (Invitrogen, Carlsbad, CA) was applied for 10 minutes. Coverslips were applied to slides using fluorescent mounting medium (Golden Bridge International, Inc., Mukilteo, WA), and tissues were visualized using a confocal microscope (Zeiss LSM 510 Meta, Karl Zeiss) equipped with a C-Apochromat 63×/1.20 W Korr UV-VIS-IR M27 water immersion objective.

### Western blot analysis

Lung homogenates or cell lysates were subjected to denaturating SDS-PAGE, followed by electroblotting and immunoblotting for anti-ATF4, anti-GRP78, anti-CHOP (Santa Cruz Biotechnology, Dallas, TX), anti-ATF6α, anti-IRE1 (Enzo Life Sciences, Farmingdale, USA), anti-XBP-1 (Novus Biologicals, Littleton, CO), anti-eIF2α, anti-phospho eIF2α, anti-phospho PERK, anti-PERK, anti-phospho AKT(Thr308), anti-phospho p70S6K (Cell Signaling Technology, Danvers, MA) or anti-phospho IRE1 (Abcam, Cambridge, MA). Blots were developed using corresponding HRP-conjugated secondary antibodies and detected using a chemiluminescent system (Amersham ECL Plus; GE Healthcare, Piscataway, NJ). Band intensities were quantified with a LAS-1000 plus system (Fuji Film, Japan).

### Lentiviral vector production and cell infection

The shRNA expression plasmids and bacterial clones for ATF6 (TRCN0000321327), IRE1 (TRCN00008427) and PERK (TRCN0000028772) were provided by the RNAi Core Facility, Academia Sinica (Taipei, Taiwan). Subconfluent fibroblast cells were infected with lentivirus in the presence of 8 μg/ml polybrene (Sigma- Aldrich). Culture medium was replaced with fresh growth medium containing puromycin (4 μg/ml) 24 hours later. Puromycin selection was performed 48 hours after infection.

### Total collagen assay

The total collagen content was measured using a total assay kit. (BioVision, Mountain View, CA). Briefly, tissue frozen in liquid N2 were dried to a constant weight and hydrolysed in 6 N HCl. 10 μL of hydrolysate was transferred to a 96-well plate and evaporated to dryness under vacuum. Data was applied following the kit protocol.

### Measurement of pulmonary function

Pulmonary function, also referred to as “airway responsiveness” *in vivo*, was measured in unrestrained mice using barometric whole body plethysmography (Buxco1; EMKA Technologies, Paris, France). Enhanced pause (Penh) values were calculated as an index of *in vivo* airway obstruction.

### Statistical analysis

All experiments were performed at least three times, and results were expressed as mean ± SD. Data significance was determined using Excel software (Microsoft Corporation). Statistical analysis was performed using one-way ANOVA for comparison of data from different treatment groups. *P* values less than 0.05 were considered statistically significant.

## Electronic supplementary material

Supplmentary figures

## References

[1] King JT, Pardo A, Selman M (2011). Idiopathic pulmonary fibrosis. Lancet.

[2] Wuyts WA (2013). The pathogenesis of pulmonary fibrosis: a moving target. Eur Respir J.

[3] Toonkel RL, Hare JM, Matthay MA, Glassberg MK (2013). Mesenchymal stem cells and idiopathic pulmonary fibrosis. Am J Respir Crit Care Med.

[4] Chen J, Stubbe J (2005). Bleomycins: Towards better therapeutics. Nature Rev.

[5] Moore BB, Hogaboam CM (2008). Murine models of pulmonary fibrosis. Am J Physiol.

[6] Moeller A, Ask K, Warburton D, Gauldie J, Kolb M (2008). The bleomycin animal model: a useful tool to investigate treatment options for idiopathic pulmonary fibrosis. Int J Biochem Cell Biol.

[7] Kaufman RJ (1999). Stress signaling from the luman of the endoplasmic reticulum: Coordination of gene transcriptional and translational controls. Genes Dev.

[8] Schroder M, Kaufman RJ (2005). The mammalian unfolded protein response. Annu Rev Biochem.

[9] Wu J, Kaufman RJ (2006). From acute ER stress to physiological roles of the unfolded protein response. Cell Death Differentiation.

[10] Ron D, Walter P (2007). Signal integration in the endoplasmic reticulum unfolded protein response. Nat Rev Mol Cell Biol.

[11] Kohno K (2007). How transmembrane proteins sense endoplasmic reticulum stress. Antioxid Redox Signal.

[12] Yoshida H, Matsui T, Yamamoto A, Okada T, Mori K (2001). XBP1 mRNA is induced by ATF6 and spliced by IRE1 in response to ER stress to produce a highly active transcription factor. Cell.

[13] Harding HP, Zhang Y, Bertolotti A, Zeng H, Ron D (2000). Perk is essential for translational regulation and cell survival during the unfolded protein response. Mol Cell.

[14] Hosoi T, Ozawa K (2010). Endoplasmic reticulum stress in disease: mechanisms and therapeutic opportunities. Clin Sci.

[15] Zhang K, Kaufman RJ (2008). From endoplasmic-reticulum stress to the inflammatory response. Nature.

[16] Baek HA (2012). Involvement of endoplasmic reticulum stress in myofibroblastic differentiation of lung fibroblasts. Am J Respir Cell Mol Biol.

[17] Zhao H (2014). Melatonin inhibits endoplasmic reticulum stress and epithelial-mesenchymal transition during bleomycin-induced pulmonary fibrosis in mice. PLOS ONE.

[18] Chang F (2003). Involvement of PI3K/Akt pathway in cell cycle progression, apoptosis, and neoplastic transformation: a target for cancer chemotherapy. Leukemia.

[19] Lu Y (2010). Phosphatidylinositol-3-kinase/Akt regulates bleomycin-induced fibroblast proliferation and collagen production. Am J Respir Cell Mol Biol.

[20] Tanaka (2015). The exacerbating roles of CCAAT/enhancer-binding protein homologous protein (CHOP) in the development of bleomycin-induced pulmonary fibrosis and the preventive effects of tauroursodeoxycholic acid (TUDCA) against pulmonary fibrosis in mice. Pharmacol Res.

[21] Xia H (2010). Pathologic caveolin-1 regulation of PTEN in idiopathic pulmonary fibrosis. Am J Pathol.

[22] Nho RS, Hergert P (2014). IPF fibroblasts are desensitized to type I collagen matrix-induced cell death by suppressing low autophagy via aberrant Akt/mTOR kinases. PLoS One.

[23] Qin L, Wang Z, Tao L, Wang Y (2010). ER stress negatively regulates AKT/TSC/mTOR pathway to enhance autophagy. Autophagy.

[24] Thon M, Hosoi T, Yoshii M, Ozawa K (2014). Leptin induced GRP78 expression through the PI3K-mTOR pathway in neuronal cells. Sci Rep.

