# Supplementary material for: Involvement of ER stress, PI3K/AKT activation, and lung fibroblast proliferation in bleomycin-induced pulmonary fibrosis
Source: Sci Rep. 2017 Oct 27;7:14272. doi: 10.1038/s41598-017-14612-5 (PMC5660192; doi:10.1038/s41598-017-14612-5)

Involvement of ER stress, PI3K/AKT activation, and lung fibroblast proliferation in bleomycin-induced pulmonary fibrosis

Han-Shui Hsu[1,2*], Chen-Chi Liu[3], Jiun-Han Lin[1,2], Tien-Wei Hsu[1,2], Jyuan-Wei Hsu[1,2] Kelly Su[1,2], Shih-Chieh Hung[4,5*]

Authors' Affiliations:

[1]Division of Thoracic Surgery, Department of Surgery, Taipei Veterans General Hospital; [2]Institute of Emergency and Critical Care Medicine, National Yang-Ming University School of Medicine; [3]Department of Emergency, Taipei Veterans General Hospital, National Yang-Ming University School of Medicine; [4]Institute of Biomedical Sciences, Academia Sinica, Taipei, Taiwan; [5]Integrative Stem Cell Center, Department of Orthopedics, China Medical University Hospital, Taichung 40447, Taiwan; [6]Graduate Institute of New Drug Development, Biomedical Sciences, China Medical University, Taichung 40402, Taiwan

Supplementary Figures

Figure S1

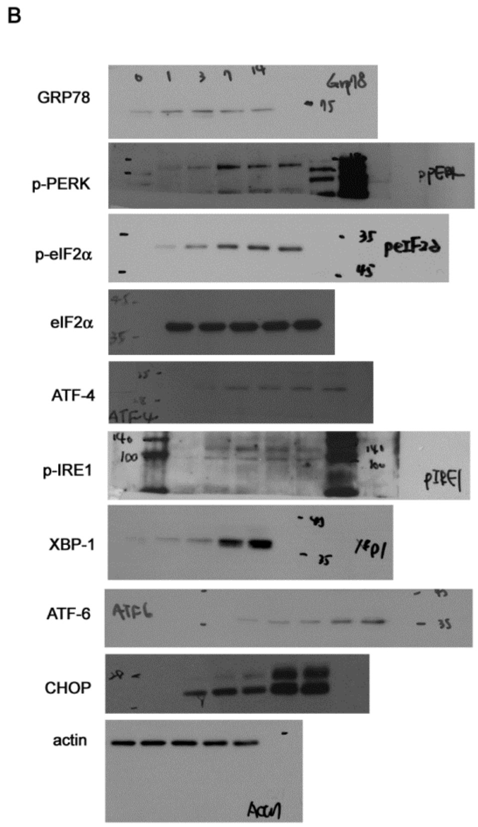

Figure S2

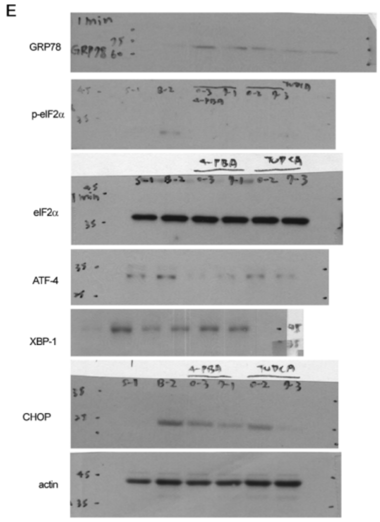

Figure S3A

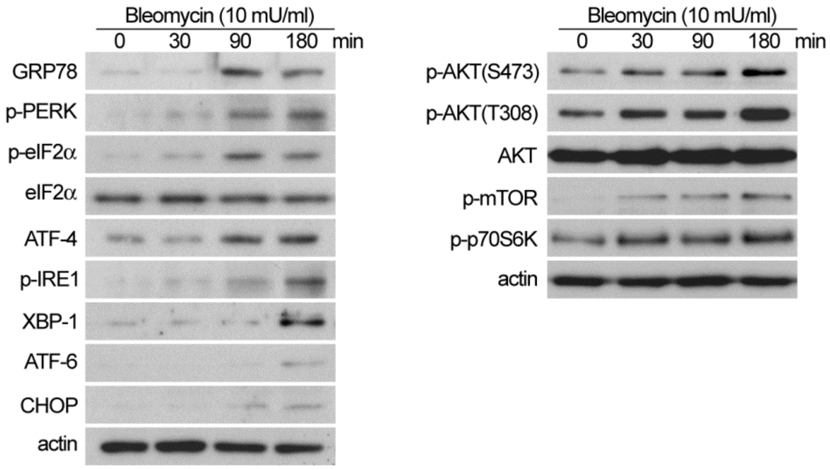

Figure S3B

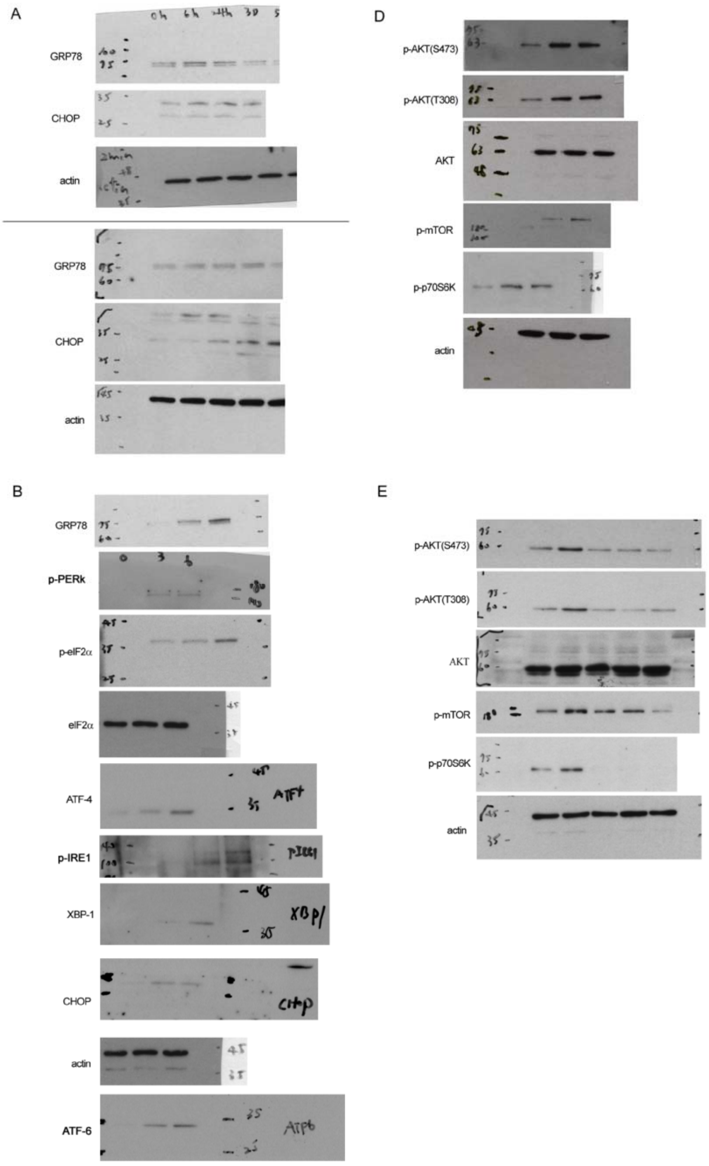

Figure S3C

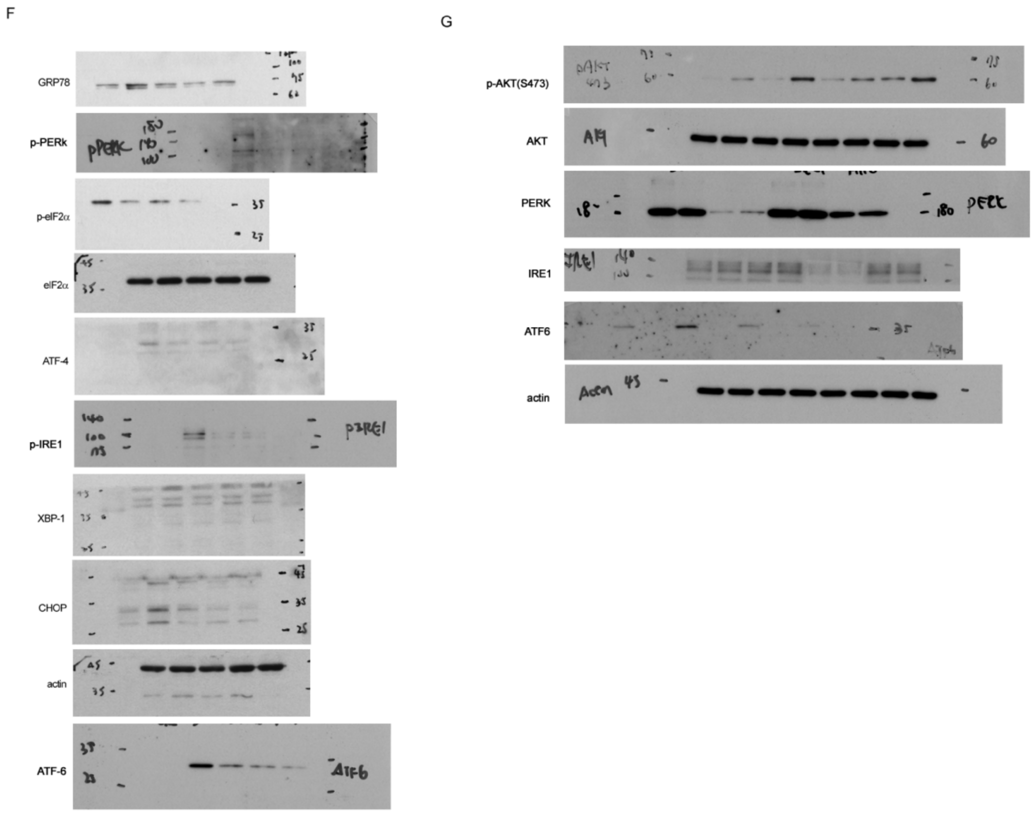

Figure S4

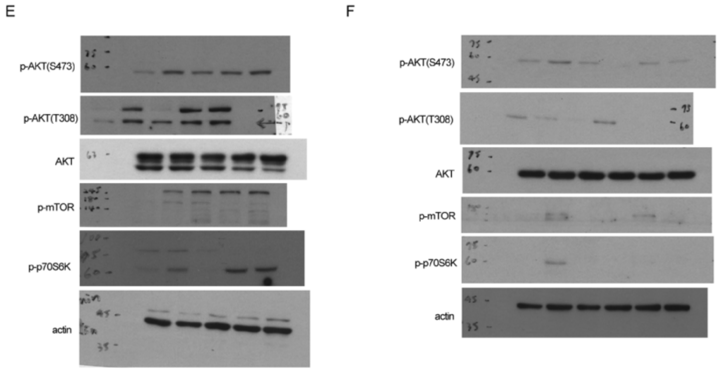

Figure S5

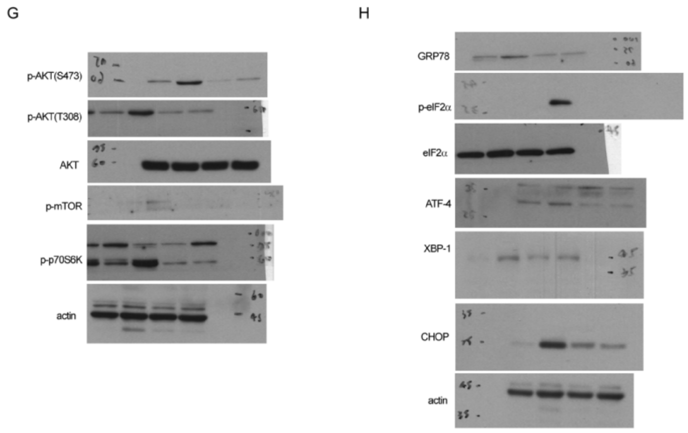

Figure S6

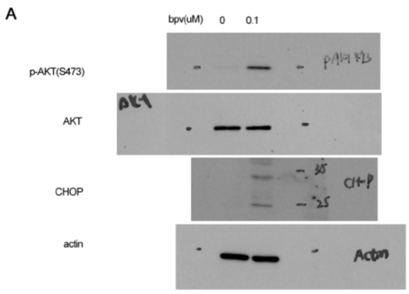

Supplement: Supplementary file 1 — Supplmentary figures [file 41598_2017_14612_MOESM1_ESM.pdf]
